# Bioinformatic analysis identifies potentially key differentially expressed genes in oncogenesis and progression of clear cell renal cell carcinoma

**DOI:** 10.7717/peerj.8096

**Published:** 2019-11-26

**Authors:** Haiping Zhang, Jian Zou, Ying Yin, Bo Zhang, Yaling Hu, Jingjing Wang, Huijun Mu

**Affiliations:** 1 Department of Derma Science Laboratory, Wuxi NO.2 People’s Hospital affiliated to Nanjing Medical University, Wuxi, Jiangsu, China; 2 Center of Clinical Research, The Affiliated Wuxi People’s Hospital of Nanjing Medical University, Wuxi, Jiangsu, China; 3 Wuxi Institute of Translational Medicine, Wuxi, Jiangsu, China

**Keywords:** Bioinformatic analysis, Renal cell carcinoma, Differentially expressed genes, Protein-protein interaction

## Abstract

Clear cell renal cell carcinoma (ccRCC) is one of the most common and lethal types of cancer within the urinary system. Great efforts have been made to elucidate the pathogeny. However, the molecular mechanism of ccRCC is still not well understood. The aim of this study is to identify key genes in the carcinogenesis and progression of ccRCC. The mRNA microarray dataset GSE53757 was downloaded from the Gene Expression Omnibus database. The GSE53757 dataset contains tumor and matched paracancerous specimens from 72 ccRCC patients with clinical stage I to IV. The linear model of microarray data (limma) package in R language was used to identify differentially expressed genes (DEGs). The protein–protein interaction (PPI) network of the DEGs was constructed using the search tool for the retrieval of interacting genes (STRING). Subsequently, we visualized molecular interaction networks by Cytoscape software and analyzed modules with MCODE. A total of 1,284, 1,416, 1,610 and 1,185 up-regulated genes, and 932, 1,236, 1,006 and 929 down-regulated genes were identified from clinical stage I to IV ccRCC patients, respectively. The overlapping DEGs among the four clinical stages contain 870 up-regulated and 645 down-regulated genes. The enrichment analysis of DEGs in the top module was carried out with DAVID. The results showed the DEGs of the top module were mainly enriched in microtubule-based movement, mitotic cytokinesis and mitotic chromosome condensation. Eleven up-regulated genes and one down-regulated gene were identified as hub genes. Survival analysis showed the high expression of CENPE, KIF20A, KIF4A, MELK, NCAPG, NDC80, NUF2, TOP2A, TPX2 and UBE2C, and low expression of ACADM gene could be involved in the carcinogenesis, invasion or recurrence of ccRCC. Literature retrieval results showed the hub gene NDC80, CENPE and ACADM might be novel targets for the diagnosis, clinical treatment and prognosis of ccRCC. In conclusion, the findings of present study may help us understand the molecular mechanisms underlying the carcinogenesis and progression of ccRCC, and provide potential diagnostic, therapeutic and prognostic biomarkers.

## Introduction

Renal cell carcinoma (RCC) is a heterogeneous group of cancers, and is one of the 10 most common cancers in the world. Based on the histopathological and molecular characterization of RCC (Hes, 2014; Moch et al., 2016), clear cell RCC (ccRCC), papillary RCC and chromophobe RCC are the major subtypes with ≥5% incidence (Cancer Genome Atlas Research Network, 2013; Cancer Genome Atlas Research Network et al., 2016; Chen et al., 2016). Clear cell RCC is the most prevalent subtype and accounts for about 80% of all RCC (Hsieh et al., 2017; Shenoy & Pagliaro, 2016). Although somatic gene mutations, including VHL, BAP-1, PBRM-1, KDM5C, SETD2, and MTOR genes (Chow, Dong & Devesa, 2010; Ricketts et al., 2018) are involved in the pathogenesis of ccRCC, the molecular mechanism of ccRCC is still not fully elucidated. Moreover, up to one-third of RCC patients have already presented with primary metastases at the time of diagnosis (Fujioka et al., 2012). Therefore, it is important to explore the molecular mechanisms of RCC and find effective biomarkers for early diagnosis. In the last decade, the high-throughput analysis platform for gene expression, such as microarray technology, has been widely used to obtain genetic alteration during tumorigenesis. Bioinformatics is a study field that uses computation to extract knowledge from biological data. It includes the collection, retrieval, manipulation and modeling of data for analysis, visualization or prediction through the algorithms and software. Bioinformatics analysis can help us identify differentially expressed genes (DEGs) and functional pathways related to the carcinogenesis and progression of cancer.

In the present study, mRNA microarray dataset from Gene Expression Omnibus (GEO) was downloaded and analyzed to obtain DEGs between ccRCC and paracancerous tissue. Afterwards, protein-protein interaction (PPI) network, gene ontology (GO) and kyoto encyclopedia of genes and genomes (KEGG) pathway enrichment analyses were performed, which help us understand the molecular mechanism of carcinogenesis and progression. In summary, in total of eleven up-regulated and one down-regulated genes were identified as hub gene, which may be candidate biomarkers for ccRCC.

## Materials and Methods

### Microarray data

The gene expression profile of GSE53757 was downloaded from the Gene Expression Omnibus (GEO) database. GSE53757, which is based on Affymetrix GPL570 platform (Affymetrix Human Genome U133 Plus 2.0 Array), was submitted by Copland et al. (Von Roemeling et al., 2014). The GSE53757 dataset contained 144 samples, including 72 ccRCC samples (Stage I 24, stage II 19, stage III 14 and stage IV 15 cases) and 72 matched normal kidney tissue.

### Affymetrix microarray data processing

Raw data (CEL file) were read by an Affy package (http://bioconductor.org/packages/release/bioc/html/affy.html) of R (version 3.4.4; http://r-project.org/). Chip data preprocessing includes background correction, data normalization, combining normal and tumor group data, ID transform gene symbol, and probe supplemental missing value. The data normalization was conducted using a robust multi-array average analysis method (Hochreiter, Clevert & Obermayer, 2006). Probe supplemental missing value was performed using k-nearest neighbor method (Sim, Kim & Lee, 2005). When multiple probes were mapped to the same gene ID, the mean expression of those probes was calculated. The linear models of microarray data (limma) package (http://bioconductor.org/packages/release/bioc/html/limma.html) in R language was used to identify DEGs between ccRCC and normal samples. Only genes with an adjusted *p* < 0.05 and —log_2_FC—>1 were selected as DEGs (where FC = fold change).

### KEGG and GO enrichment analyses of DEGs

The Database for annotation, visualization and integrated discovery (DAVID, https://david.ncifcrf.gov/tools.jsp) is an online program that provides a comprehensive set of functional annotation tools for researchers to understand biological meaning behind plenty of genes(Sherman et al., 2007). Gene ontology (GO) and kyoto encyclopedia of genes and genomes (KEGG) pathway enrichment analysis were performed for identified DEGs using DAVID database. *P* < 0.05 was set as the cut-off criterion.

### Construction of protein–protein interaction network and module analysis

The functional interactions between proteins may provide insights into the molecular mechanism of cellular processing. In this study, protein-protein interaction (PPI) networks were constructed for the DEGs using the STRING database (https://string-db.org/) (Szklarczyk et al., 2015), which provides a critical integration of PPIs, including known and predicted interactions. The interacting pairs with a combined score >0.7 (high confidence) were selected for the PPI network construction. Subsequently, PPI network was visualized using Cytoscape software (3.6.1) (Demchak et al., 2014). The molecular complex detection (MCODE, version 1.5.1) algorithm is a Cytoscape plugin (Bader & Hogue, 2003), which clusters a given network based on topology to find densely connected regions. The most significant module in the PPI networks was screened using MCODE with score >5, degree cut-off = 10, node score cut-off = 0.2, k-core = 2 and max depth = 100.

The functional enrichment analysis of genes in each module was performed by DAVID.

### Hub genes selection

In this study, Cytoscape plugin cytoHubba is used for selected hub genes. CytoHubba is a novel Cytoscape plugin for exploring important nodes in an interactome network by several topological algorithms, including degree, edge percolated component (EPC), maximum neighborhood component (MNC) and maximal clique centrality (MCC) (Chin et al., 2014). The overlapped genes were selected as candidate hub gene in the four algorithms of cytoHubba.

### Validation of the hub genes using MEXPRESS database and survival analysis

MEXPRESS (http://mexpress.be/) is a data tool designed for the easy visualization of the Cancer Genome Atlas (TCGA) expression and clinical data, as well as the relationships between them (Koch et al., 2015). To confirm the reliability of the hub genes, we used the MEXPRESS to validate the expression level of the candidate hub genes in ccRCC. The overall survival analyses of hub genes were performed using gene expression profiling interactive analysis (GEPIA, http://gepia.cancer-pku.cn/index.html) online platform, which based on TCGA datasets.

### Validation of the hub genes by quantitative real-time PCR

Validation of selected hub gene was conducted using quantitative real-time PCR. Forty-four primary ccRCC and paired normal tissues were obtained from the operative specimens. The patients consisted of 6 females and 38 males, with age from 17 to 85 years (55.88 ± 13.36). All the protocols conformed to the ethical guidelines of the 1975 Helsinki Declaration, and were approved by the ethics committee on clinical new technologies and scientific research of Wuxi People’s Hospital (Permit number: KS00025). The written informed consents were obtained before the specimens were collected. The total RNA was extracted using Trizol (Invitrogen GIBco) following the manufacturer’s instructions. First-strand cDNA was synthesized using MMLV reverse transcriptase (Promega, America) and random primers according to the manufacturer’s instructions. 20 µL PCR reaction system contains 2 µL 25 mM/L MgCl_2_, 5mM/ µL sense and antisense primers (1.0 µL each), 0.4 µL 10 mM/L dNTP, 1.0 µL Evagreen (Biotium), 2.0 µL 5*PCR buffer, 2.0 µL cDNA, 0.5 Unit *Taq* DNA polymerase (Promega). Quantitative real-time PCR analysis was conducted on Lightcycler 480 (Roche, Switzerland) with the following PCR profile: predenaturation at 95 °C for 5 min; 40 cycles of denaturation at 95 °C for 15 s, annealing at 60 °C for 10 s and elongation at 72 °C for 15 s. The expression changes of candidate hub genes between malignant and adjacent normal tissues were calculated as 2^−ΔΔ*CT*^ using the comparative ΔΔCT method.

### Statistical analysis

The student’s *t*-test was used to check whether real-time PCR expression data in malignant and adjacent normal tissues differ significantly. Gene with *p* < 0.05 and >2-fold change was considered to be significantly different.

## Results

### Identification of DEGs

**Figure 1:**
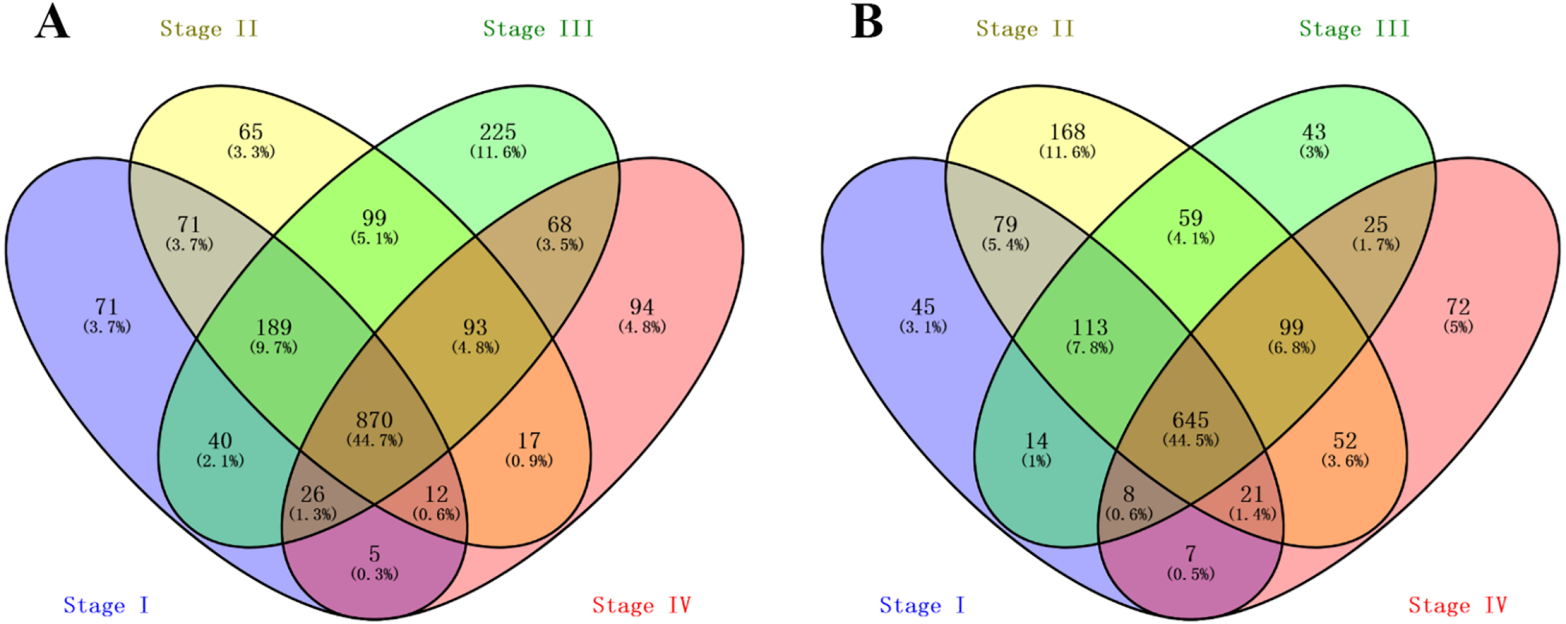
Venn Diagram showing the numbers of overlap differentially expressed genes in the four stages clear cell renal cell carcinoma: (A) the numbers of upregulated genes in four stages of ccRCC patients; (B) the numbers of downregulated genes in four stages of ccRCC patients.

A total of 1,284, 1,416, 1,610 and 1,185 up-regulated genes, and 932, 1,236, 1,006 and 929 down-regulated genes were identified between normal and malignant tissue samples from stage I, II, III and IV ccRCC patients, respectively. The overlapping DEGs across all stages contained 870 up-regulated and 645 down-regulated genes, as shown in the Venn diagram (Figs. 1A and 1B).

### KEGG and GO enrichment analyses of DEGs

To gain further insight into the identified DEGs, functional and pathway enrichment analyses were conducted using DAVID. Gene ontology (GO) enrichment analysis showed that up-regulated DEGs were mainly involved in biological processes (BP), including immune response, inflammatory response, and interferon-gamma-mediated signaling pathway, while down-regulated DEGs were significantly enriched in oxidation—reduction process, sodium ion transport and excretion process. KEGG pathway analysis showed the up-regulated DEGs were enriched in phagosome and allograft rejection, while the down-regulated DEGs were enriched in metabolic pathways and biosynthesis of antibiotics. The top five over-represented GO terms under biological process (BP), cellular component (CC) and molecular function (MF), and the main enriched pathways are shown in Table 1.

**Table 1:** Top five GO terms and significant pathways enriched by DEGs.

**Expression change**	**Category**	**Term**	**Count**	**FDR**
**Up**	BP	GO:0006955 ∼immune response	82	1.68E–27
		GO:0006954 ∼inflammatory response	65	4.94E–18
		GO:0060333 ∼interferon-gamma-mediated signaling pathway	25	1.81E–12
		GO:0050776 ∼regulation of immune response	37	1.17E–11
		GO:0051607 ∼defense response to virus	31	1.68 E–27
	CC	GO:0009897 ∼external side of plasma membrane	40	1.37 E–11
		GO:0005886 ∼plasma membrane	267	4.06 E–11
		GO:0009986 ∼cell surface	66	5.70 E–11
		GO:0005887 ∼integral component of plasma membrane	118	2.64 E–09
		GO:0005615 ∼extracellular space	107	6.10 E–07
	MF	GO:0004872 ∼receptor activity	32	5.21 E–06
		GO:0005102 ∼receptor binding	42	9.04 E–06
		GO:0042605 ∼peptide antigen binding	12	1.35 E–05
		GO:0005201 ∼extracellular matrix structural constituent	14	6.51 E–03
		GO:0005515 ∼protein binding	431	2.13 E–02
	PATHWAY	hsa04145:Phagosome	37	5.57 E–12
		hsa05416:Viral myocarditis	23	3.25 E–11
		hsa05330:Allograft rejection	19	5.75 E–11
		hsa05332:Graft-versus-host disease	18	8.75 E–11
		hsa05150:Staphylococcus aureus infection	22	1.07 E–10
**Down**	BP	GO:0055114 ∼oxidation–reduction process	56	1.02 E–09
		GO:0006814∼sodium ion transport	16	5.11 E–05
		GO:0007588 ∼excretion	11	2.49 E–04
		GO:0055078 ∼sodium ion homeostasis	7	6.67 E–04
		GO:0001657 ∼ureteric bud development	10	3.58 E–03
	CC	GO:0070062 ∼extracellular exosome	215	7.05 E–38
		GO:0016324 ∼apical plasma membrane	44	9.64 E–15
		GO:0016323 ∼basolateral plasma membrane	31	4.53 E–11
		GO:0005759 ∼mitochondrial matrix	38	1.05E–08
		GO:0005782 ∼peroxisomal matrix	11	0.001373
	MF	GO:0003824 ∼catalytic activity	23	1.23E–04
		GO:0009055 ∼electron carrier activity	16	1.42E–04
		GO:0050660 ∼flavin adenine dinucleotide binding	13	7.38E–04
		GO:0016491 ∼oxidoreductase activity	22	1.50E–03
		GO:0030170 ∼pyridoxal phosphate binding	12	1.64E–03
	PATHWAY	hsa01100:Metabolic pathways	111	3.54E–16
		hsa01130:Biosynthesis of antibiotics	31	1.14E–06
		hsa00280:Valine, leucine and isoleucine degradation	13	2.41E–04
		hsa01200:Carbon metabolism	18	2.50E–03
		hsa00071:Fatty acid degradation	11	5.18E–03

### PPI network construction and modules analysis

**Figure 2:**
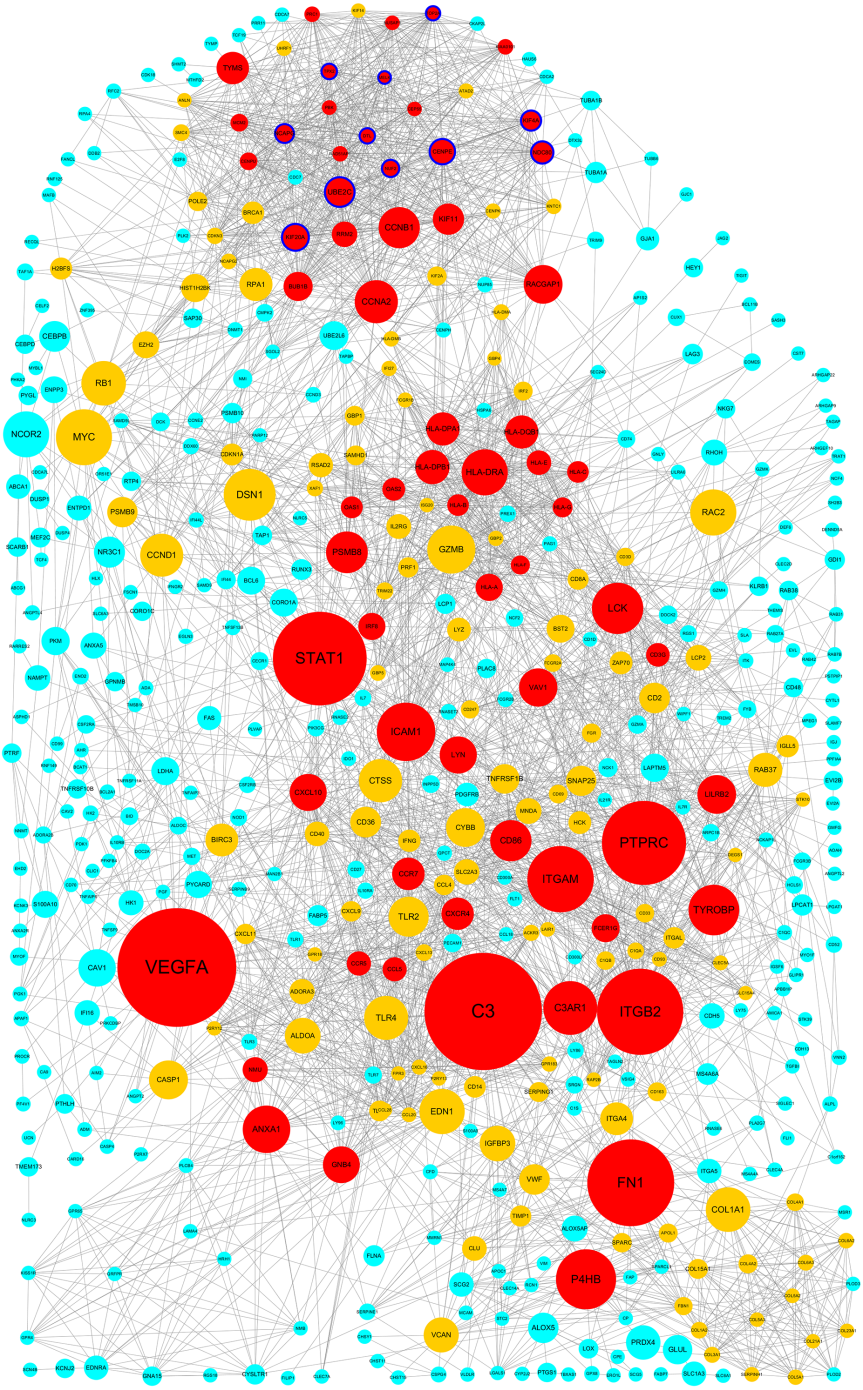
Protein–protein interaction network among up–regulated genes detected in ccRCC** (GSE53757 dataset). Nodes represent genes and edges indicate interaction between proteins. Nodes are colored based on the number of degrees: 1∼15 (light blue), 16∼30 (yellow) and 30∼57(red). Node size indicates betweenness centrality values. Hub genes are represented with a thicker blue border.

**Figure 3:**
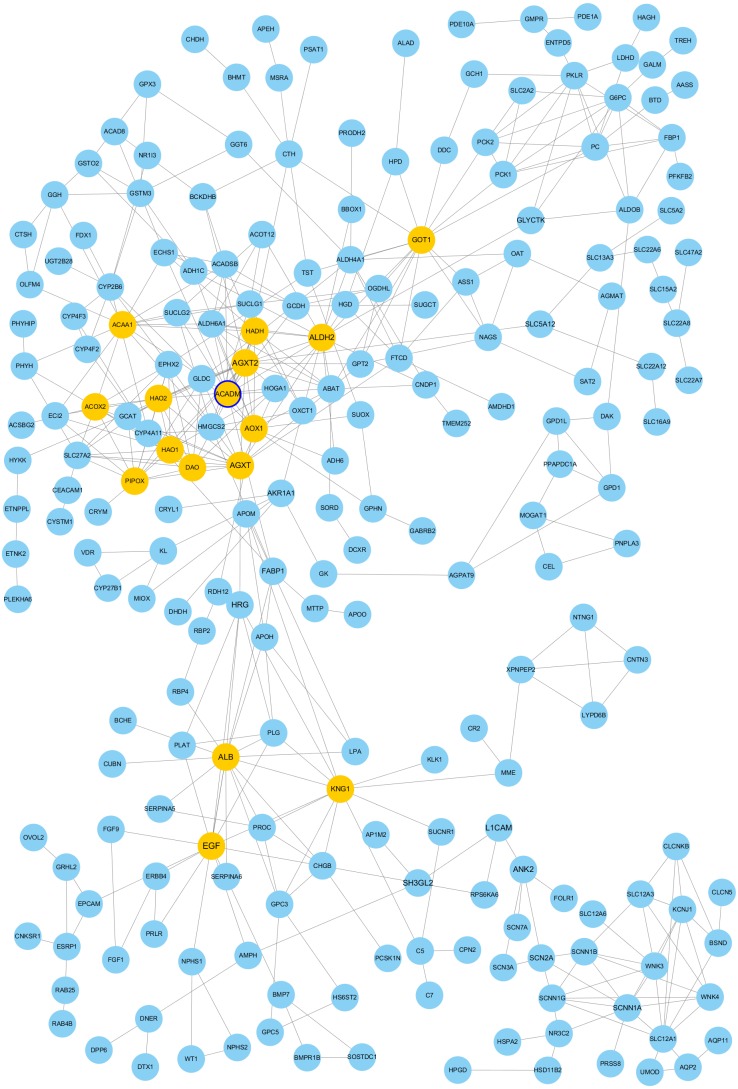
Protein-protein interaction network among down–regulated genes detected in ccRCC** (GSE53757 dataset). Nodes represent genes and edges indicate interaction between proteins. Nodes are colored based on the number of degrees: 1∼9 (light blue) and 10∼19 (yellow). Node size indicate betweenness centrality values. Hub genes are represented with a thicker blue border.

**Figure 4:**
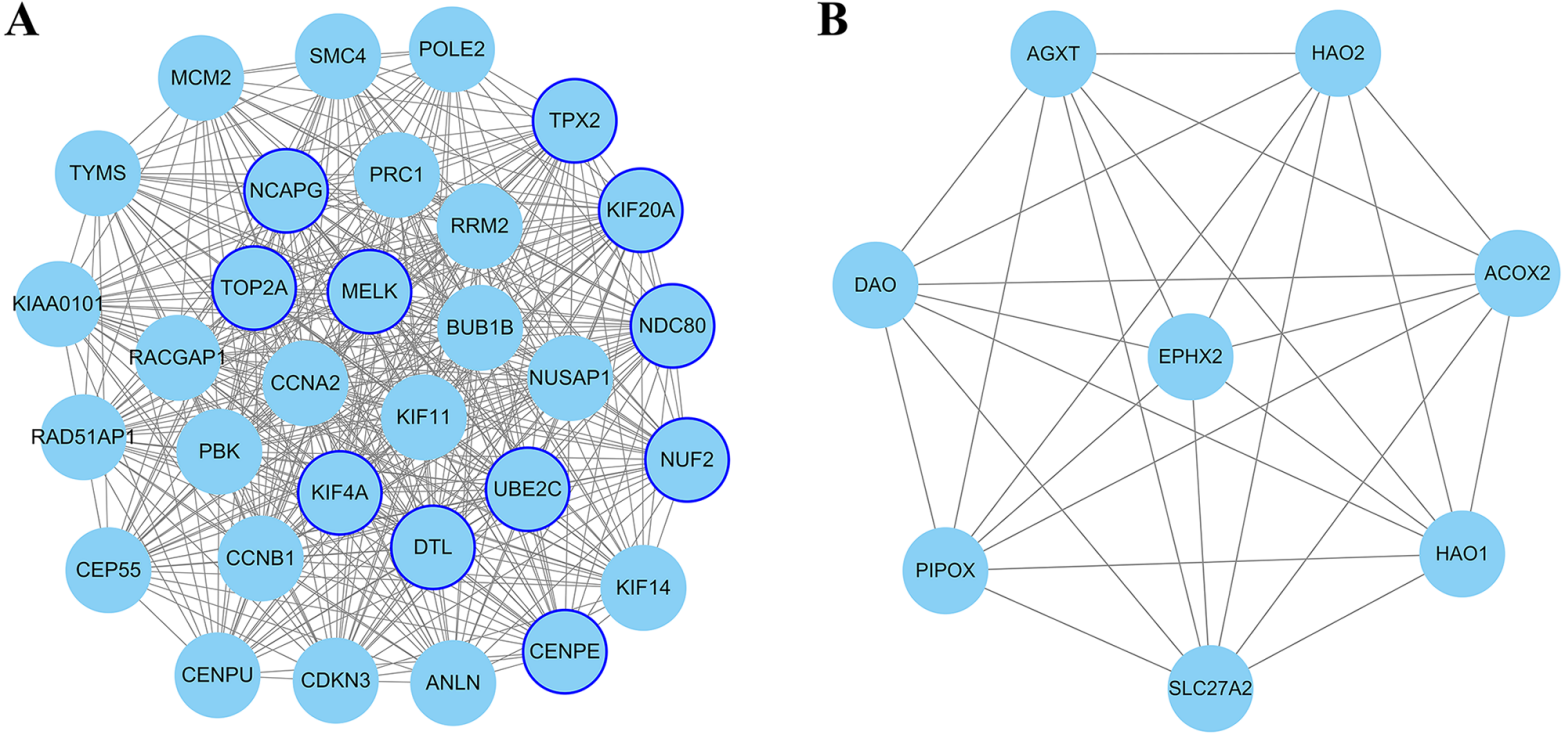
The most significant module of differentially expressed genes (DEGs). (A) The most significant module was obtained from PPI network of up-regulated DEGs with 31 nodes and 432 edges. (B) The most significant module was obtained from PPI network down-regulated DEGs with eight nodes and 28 edges. The thicker blue border represents the hub gene.

The PPI network of DEGs was constructed using Cytoscape. The PPI networks of up and down-regulated DEGs consisted of 543 nodes and 3,590 edges, 301 nodes and 507 edges, respectively (Figs. 2 and 3). Using the MCODE app, the most significant module with highest score in the PPI network for ccRCC was detected. The most significant module from up-regulated DEGs PPI network contains 31 nodes and 432 edges (Fig. 4A), and eight nodes and 28 edges form down-regulated DEGs PPI network (Fig. 4B). The enrichment analysis of DEGs in the modules was also carried out with DAVID. The results showed that these genes were mainly enriched in microtubule-based movement, mitotic cytokinesis and mitotic chromosome condensation.

### Hub gene selection

In the present study, Cytoscape plugin cytoHubba is used for selected hub genes. Degree is equal to or greater than 10 as the threshold of the hub gene. In this PPI network, the degree of 267 up-regulated and 16 down-regulated genes is equal to or greater than 10. Twenty-one genes appeared in the top 50 up-regulated genes list in terms of degree, EPC, MNC and MCC, simultaneously. Similarly, four genes appeared in the top 10 down-regulated genes list. These genes were selected as candidate hub gene which overlapped in the four algorithms of cytoHubba.

### Hub genes validation using MEXPRESS database

**Figure 5:**
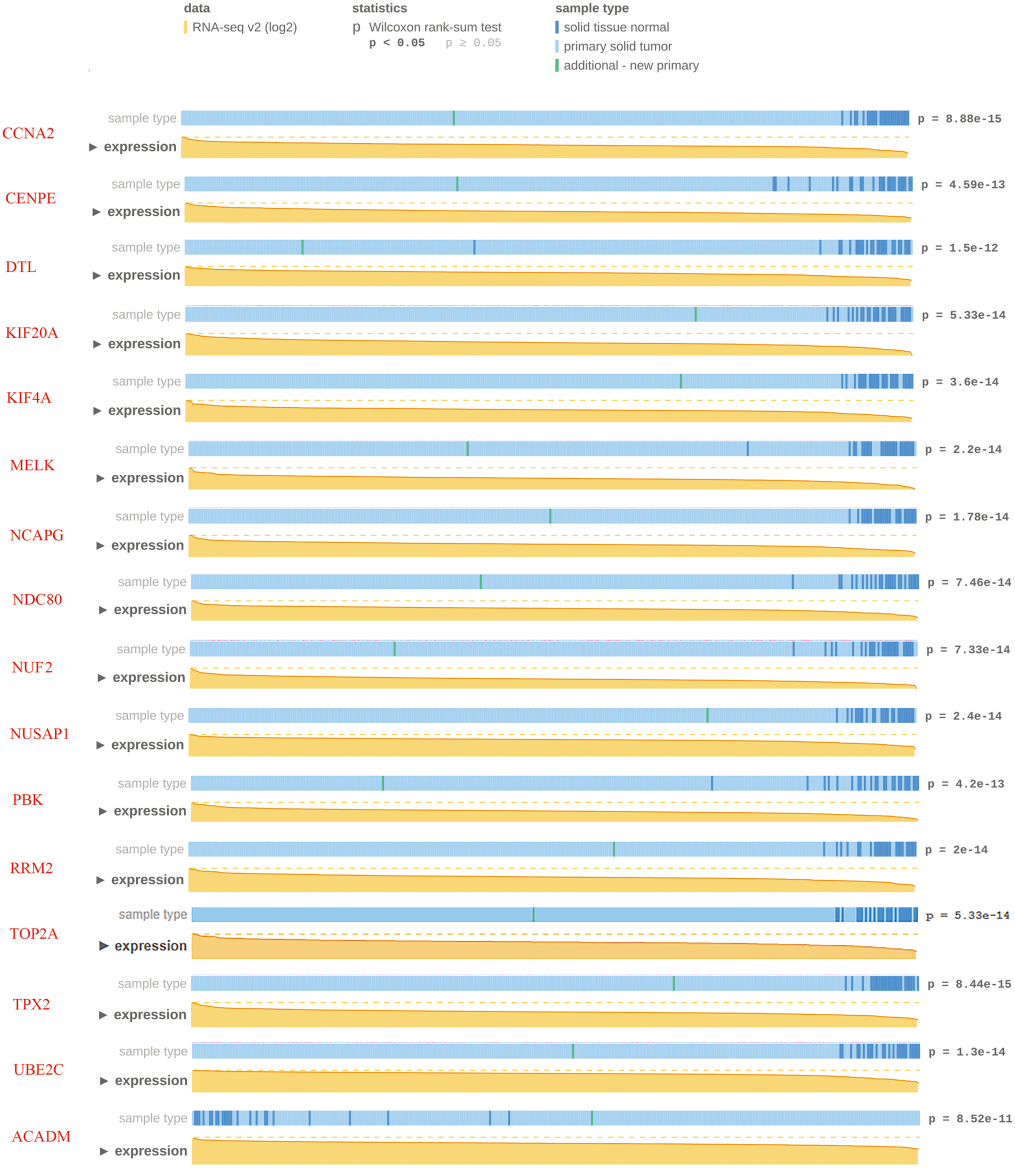
Visualization of the TCGA data for candidate hub differentially expressed genes using MEXPRESS. Visualization of the TCGA data for candidate hub differentially expressed genes in clear cell renal cell carcinoma. The height of the orange line represents the logarithm of the level 3 RNA-sequencing data in TCGA (normalized RNASeqV2 values per gene). The expression data forms the basis of the whole plot, because the samples are ranked based on their expression value for the gene we selected with the highest expression on the left side and the lowest on the right.

To confirm the reliability of the hub genes, we used MEXPRESS to validate the expression level of candidate hub genes in ccRCC. This TCGA dataset contains RNA-seq v2 (log2) from 343 clinical ccRCC specimens. The data showed that except for two down-regulated candidate hub genes, the expression of other candidate hub genes were consistent with the TCGA database (*p* < 0.05). Nevertheless, MEXPRESS visualization showed that for some genes with significant differences in expression, the distribution of tumor specimens was not highly concentrated. In order to ensure the reliability of hub gene selection, we screened 15 up-regulated and one down-regulated genes with *p* < 1.0E–10 for further verification (Fig. 5).

### Validation of hub gene by quantitative real-time PCR

Quantitative real-time PCR was used to verify the expression profiles of 15 up-regulated genes and one down-regulated gene in 44 patients with ccRCC. Table 2 lists the primers for real-time PCR detection of 16 candidate hub genes. Table 3 shows mRNA expression of candidate hub genes between tumor and paracancerous tissue. The data showed that the expression of 11 up-regulated genes and one down-regulated gene had significant difference between tumors and adjacent tissues, but not for CCNA2, NUSAP1, PBK and RRM2 gene. Therefore, a total of 11 up-regulated genes and one down-regulated gene were identified as the hub genes. The names, abbreviations and functions of hub genes are shown in Table 4. Table 5 shows the average fold change of hub genes between malignant and normal tissue samples from chip dataset.

**Table 2:** Primers of candidate hub genes for real-time PCR assay.

**Gene**	**Gene ID**		**Sequence**	**Amplicon (bp)**
CCNA2	890	Sense	5′-CTCTACACAGTCACGGGACAAAG-3′	120
		Antisense	5′-CTGTGGTGCTTTGAGGTAGGTC-3′	
CENPE	1,062	Sense	5′-GGAGAAAGATGACCTACAGAGGC-3′	111
		Antisense	5′-AGTTCCTCTTCAGTTTCCAGGTG-3′	
DTL	51,514	Sense	5′-CCAGCCTTAGTCCAGATGACCA-3′	114
		Antisense	5′-GAGAATGACCCAGGAGCACAGT-3′	
KIF20A	10,112	Sense	5′-CAAGAGGCAGACTTTGCGGCTA-3′	130
		Antisense	5′-GCTCTGGTTCTTACGACCCACT-3′	
KIF4A	24,137	Sense	5′- GTGGAGCAAGAAGCCCAAGT-3′	97
		Antisense	5′-TAGACATCTGCGCTTGACGG-3′	
MELK	9,833	Sense	5′-TCCTGTGGACAAGCCAGTGCTA-3′	102
		Antisense	5′-GGGAGTAGCAGCACCTGTTGAT-3′	
NCAPG	64,151	Sense	5′-ACAGGATTTTAATCGGGCATCAG-3′	138
		Antisense	5′-TGCAATGTTTCAGCATCATTCTTCT-3′	
NDC80	10,403	Sense	5′-CTGACACAAAGTTTGAAGAAGAGG-3′	128
		Antisense	5′-TAAGGCTGCCACAATGTGAGGC-3′	
NUF2	83,540	Sense	5′-TGGAGACTCAGTTGACTGCCTG-3′	135
		Antisense	5′-ATTTGGTCCTCCAAGTTCAGGCT-3′	
NUSAP1	51,203	Sense	5′-CTGACCAAGACTCCAGCCAG-3′	114
		Antisense	5′-AGCAGAATTCCCCGTGATGG-3′	
PBK	55,872	Sense	5′-AATATGACTGTGACTGACCCTGA-3′	83
		Antisense	5′-ACACCATTCTCCTCCACAGC-3′	
RRM2	6,241	Sense	5′-CTGGCTCAAGAAACGAGGACTG-3′	132
		Antisense	5′-CTCTCCTCCGATGGTTTGTGTAC-3′	
TOP2A	7,153	Sense	5′-GTGGCAAGGATTCTGCTAGTCC-3′	135
		Antisense	5′-ACCATTCAGGCTCAACACGCTG-3′	
TPX2	22,974	Sense	5′-GACTTCCACTTCCGCACAGA-3′	122
		Antisense	5′-TTAGTCACTCGGGCAGGAGA-3′	
UBE2C	11,065	Sense	5′-TGATGTCTGGCGATAAAGGGA-3′	121
		Antisense	5′-AGCGAGAGCTTATACCTCAGG-3′	
ACADM	34	Sense	5′-GCCAATCGACAACGTGAACC-3′	117
		Antisense	5′-TGCAGCCACTGGGATGATTT-3′	
GAPDH	2,597	Sense	5′-CAACTTTGGTATCGTGGAAGGACTC-3′	128
		Antisense	5′-AGGGATGATGTTCTGGAGAGCC-3′	

**Table 3:** The mRNA expression of candidate hub genes in 44 ccRCC patients using real-time PCR.

Gene	Transcript ID	Cancer tissue (*N* = 44)	Paracanceroustissue (*N* = 44)	2^−(ΔΔCT)^	t
CCNA2	NM_001237	30.56 ± 1.59	34.39 ± 1.69	1.78	1.65
CENPE	NM_001813	33.65 ± 1.57	38.30 ± 1.67	3.13	3.28^*^
DTL	NM_016448	27.18 ± 1.60	32.76 ± 1.88	5.99	4.96^*^
KIF20A	NM_005733	32.79 ± 1.62	37.54 ± 1.90	3.39	3.36^*^
KIF4A	NM_012310	28.45 ± 1.78	32.59 ± 1.50	2.20	2.26^#^
MELK	NM_014791	24.45 ± 1.55	28.78 ± 2.12	2.52	2.48^*^
NCAPG	NM_022346	28.97 ± 1.72	34.35 ± 2.63	5.23	4.01^*^
NDC80	NM_006101.3	29.65 ± 1.84	35.57 ± 2.40	7.56	5.01^*^
NUF2	NM_145697.3	28.20 ± 2.07	32.12 ± 1.62	1.90	1.73^#^
NUSAP1	NM_016359	25.48 ± 1.41	28.31 ± 1.03	0.89	0.36
PBK	NM_018492	28.66 ± 1.43	30.75 ± 1.69	0.53	1.84
RRM2	NM_001165931	24.72 ± 1.58	28.26 ± 1.71	1.02	0.06
TOP2A	NM_001067	26.74 ± 1.92	32.71 ± 2.25	7.84	5.16^*^
TPX2	NM_012112	27.22 ± 1.58	32.62 ± 1.84	5.28	4.66^*^
UBE2C	NM_007019	29.82 ± 2.38	36.08 ± 2.18	9.64	5.38^*^
ACADM	NM_000016	23.55 ± 1.34	23.99 ± 1.81	0.17	4.78^*^
GAPDH	NM_002046.7	18.01 ± 1.69	21.00 ± 1.72		

**Notes.**

All results were expressed as the Means ± SD of cycle threshold (Cq).

* *p* < 0.01.

# *p* < 0.05.

**Table 4:** Functional roles of hub genes.

**Gene**	**Full name**	**Function**
CENPE	Centromere Protein E	Required for kinetochore function and chromosome segregation in mitosis.
DTL	Denticleless E3 Ubiquitin Protein Ligase Homolog	Required for cell cycle control, DNA damage response and translesion DNA synthesis.
KIF20A	Kinesin family member 20A	Required for chromosome passenger complex (CPC)-mediated cytokinesis.
KIF4A	Kinesin Family Member 4A	Translocates PRC1 to the plus ends of interdigitating spindle microtubules during the metaphase to anaphase transition.
MELK	Maternal Embryonic Leucine Zipper Kinase	Involved in various processes such as cell cycle regulation, self-renewal of stem cells, apoptosis and splicing regulation.
NCAPG	Non-SMC Condensin I Complex Subunit G	Regulatory subunit of the condensin complex, required for conversion of interphase chromatin into mitotic-like condense chromosomes.
NDC80	Kinetochore Complex Component	Acts as a component of NDC80 complex, which is required for chromosome segregation and spindle checkpoint activity.
NUF2	NDC80 Kinetochore Complex Component	Acts as a component of NDC80 complex, which is required for chromosome segregation and spindle checkpoint activity.
TOP2A	DNA Topoisomerase II Alpha	Catalyzing the ATP dependent breakage and rejoining of double strand of DNA,
TPX2	Microtubule Nucleation Factor	Spindle assembly factor required for normal assembly of mitotic spindles.
UBE2C	Ubiquitin Conjugating Enzyme E2 C	Acts as an essential factor of the anaphase promoting complex.
ACADM	Acyl-CoA Dehydrogenase Medium Chain	Catalyzes the initial step of fatty acid beta-oxidation.

**Table 5:** Fold change of hub genes between normal and malignant tissue samples from chip dataset.

Gene symbol	LogFC (Average in four stages)	FDR (Max in four stages)
CENPE	1.32	3.24E–06
DTL	1.38	1.78E–08
KIF20A	2.27	2.42E–07
KIF4A	1.70	3.88E–06
MELK	1.32	8.26E–09
NCAPG	1.51	2.92E–08
NDC80	1.47	2.43E–04
NUF2	1.65	1.82E–06
TOP2A	1.60	1.68E–09
TPX2	1.70	3.38E–06
UBE2C	2.00	2.64E–08
ACADM	2.77	3.46E–05

**Notes.**

FC fold change FDR false discovery rate

### Survival analyses of hub genes

**Figure 6:**
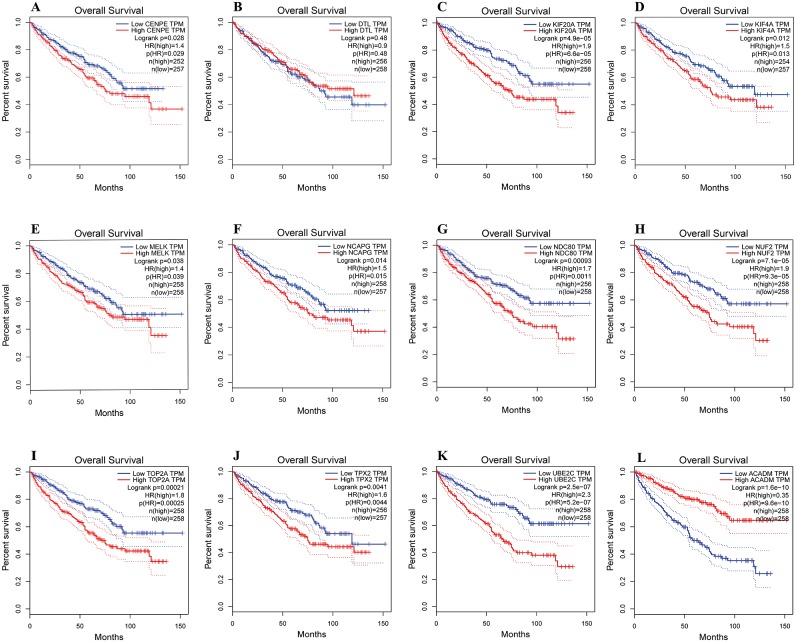
Overall survival analyses of hub genes were performed using gene expression profiling interactive analysis (GEPIA) online platform. *p* < 0.05 was considered statistically significant. Kaplan-Meier survival curve showed that 11 hub genes with high expression level (A, CENPE; C, KIF20A; D, KIF4A; E, MELK; F, NCAPG; G, NDC80; H, NUF2; I, TOP2A; J, TPX2; K, UBE2C) and a low expression gene (L, ACADM) were significantly associated with malignant outcome in ccRCC patients. High expression of DTL gene was not significantly associated with prognosis in ccRCC patients.

The overall survival analyses of hub genes were performed using Gene Expression Profiling Interactive Analysis (GEPIA, http://gepia.cancer-pku.cn/index.html) online platform, which based on TCGA datasets. The data showed the ccRCC patients with high expression of CENPE, KIF20A, KIF4A, MELK, NCAPG, NDC80, NUF2, TOP2A, TPX2 and UBE2C gene had worse overall survival (Figs. 6A, 6C–6K, *p* < 0.05). The data also showed the ccRCC patients with low expression of ACADM gene had worse overall survival (Fig. 6L, *p* < 0.01).

## Discussion

Clear cell renal cell carcinoma (ccRCC) is the most frequent form of urologic malignancy with numerous genetic alterations. The most common genetic changes associated with the development of ccRCC are the deletion of the short arm of chromosome 3. Other genetic alterations include gain of 5q, partial loss of 14q, 8p deletion, 9p loss and 7q gain (Li et al., 2017). Despite our understanding of the biology and pathophysiology of ccRCC has improved significantly over the past decade, the overall mortality of ccRCC has remained largely unchanged. The lethality of ccRCC is mainly due to the difficulty of early detection and the lack of effective treatment. Traditional chemotherapy and radiotherapy are almost ineffective in the treatment of ccRCC (Makhov et al., 2018). Therefore, potential markers for early diagnosis and effective treatment are urgently demanded. With the development of high-throughput technology nowadays, bioinformatics analysis enables us to explore the genetic alterations and identify new biomarkers in ccRCC (Batai et al., 2018), which have possible clinical applications for diagnosis, therapeutic, and prognosis.

In the present study, we analyzed the mRNA microarray dataset to obtain DEGs between ccRCC and paracancerous tissues. A total of 896 up-regulated and 653 down-regulated genes were identified in four stages of ccRCC. We constructed the PPI network of DEGs and got the most significant module. Functional annotation showed that DEGs in the modules were mainly enriched in mitotic cell cycle regulation. High expression of these genes accelerates cell cycle progression and promotes the proliferation of cancer cells (Santo, Siu & Raje, 2015). Eleven up-regulated and one down-regulated gene were identified as hub genes by validation of TCGA database and clinical specimens. All the up-regulated hub genes are located in the most significant module.

Aberrations of the mitotic cell cycle play important roles in the carcinogenesis or progression of tumors. Dysregulation of the cell cycle is recognized as a hallmark of malignancy (Hanahan & Weinberg, 2011). In the present study, we found five high expression hub genes involved in the cell cycle, including DTL, MELK, NDC80, NUF2 and UBE2C. DTL (Denticleless E3 Ubiquitin Protein Ligase Homolog) is a critical regulator of cell cycle progression and genome stability. DTL mediates the poly-ubiquitination and subsequent degradation of CDKN1A/p21 and CDT1 (Baraniskin et al., 2012; Ma et al., 2014). Overexpression of DTL is related to the poor outcome in gastric carcinoma (Kobayashi et al., 2015), breast and lung cancers (Perez-Pena et al., 2017). MELK (Maternal embryonic leucine zipper kinase) is involved in various processes such as cell cycle, apoptosis and splicing regulation. MELK is an oncogenic kinase essential for early recurrence of hepatocellular carcinoma (Xia et al., 2016). Studies have shown that MELK is a novel biomarker and a potential therapeutic target in cervical cancer (Lv et al., 2018), triple-negative breast cancer (Moreno, 2016) and gastric cancer (Zhang et al., 2016a). NDC80 (Nuclear division cycle 80) is also known as highly expressed in cancer 1 (HEC1). NDC80 is a mitotic protein that regulates cell cycle by interacting with SAC protein kinase (Ji, Gao & Yu, 2015), binding with phosphorylated spindle and kinetochore-associated protein 3 (Zhang et al., 2017). Studies have shown that NDC80 is overexpressed in colorectal cancer (Yan et al., 2018), pancreatic cancer (Meng et al., 2015) and gastric cancer (Qu et al., 2014), which may play a crucial role in carcinogenesis. NUF2 also called as CDCA1, is a member of the NDC80 complex, which plays an important role in regulating mitosis. NUF2 is a novel cancer biomarker overexpressed in lung cancer (Harao et al., 2008), colorectal cancer and pancreatic cancer (Hu et al., 2015; Kobayashi et al., 2014). Study suggests NUF2 as a novel prognostic biomarker and therapeutic target for cancers (Thang et al., 2016). UBE2C (Ubiquitin conjugating enzyme E2C) is a member of the E2 ubiquitin conjugating enzyme family. UBE2C is necessary for the destruction of mitotic cyclins and cell cycle progression, and may participate in the cancer progression (Zollner et al., 2017). UBE2C is considered to be a crucial factor upregulated in various malignancies, including breast cancer, melanoma, esophageal squamous cell carcinoma, colorectal cancer and gastric cancer (Kraft et al., 2017; Ma et al., 2018; Palumbo Jr et al., 2016; Pellino et al., 2016; Qin et al., 2017).

In recent years, kinesin motor proteins have become a potential target for cancer therapy (Huszar et al., 2009; Kaestner & Bastians, 2010). In the present study, kinesin motor protein KIF4A, KIF20A and CENPE are involved in the carcinogenesis and progression of ccRCC. KIF4A and KIF20A are members of the kinesin protein superfamily, which is microtubule-dependent molecular motor and mediates the transport of organelles, vesicles and chromosomes, as well as the movement of microtubules within the cell. In hepatocellular carcinoma (Huang et al., 2018), breast cancer (Xue et al., 2018) and prostate cancer (Huang & Gao, 2018), the up-regulation of KIF4A can predict poor prognosis. Increased expression of KIF4A is also associated with lymph node metastasis in colorectal cancer (Matsumoto et al., 2018). KIF20A expression is aberrant in various cancers, such as cervical squamous cell carcinoma (Zhang et al., 2016b), pancreatic cancer (Stangel et al., 2015) and glioma (Saito et al., 2017). Overexpression of KIF20A is correlated with poor overall survival of hepatocellular carcinoma (De Carcer et al., 2018), lung adenocarcinoma (Zhao et al., 2018) and ccRCC (Yuan et al., 2017). CENPE (Centrosome-associated protein E) is a kinesin-like motor protein that accumulates in the G2 phase of the cell cycle. CENPE plays an important role in chromosome congression, microtubule-kinetochore conjugation and spindle assembly checkpoint activation. CENPE is highly expressed in lung adenocarcinoma (Gao, Wang & Zhang, 2019), breast cancer (Yuan et al., 2018) and esophageal adenocarcinoma (Zhu et al., 2019). The study showed the overall survival rate of NSCLC patients with high expression of CENPE was poor (Hao & Qu, 2019).

Microtubule-associated proteins are involved in various cellular functions, such as motility, intracellular trafficking and mitotic spindle formation. Drugs that interfere with microtubule function can cause cell cycle arrest or apoptosis and prevent cells from mitosis. Owing to its critical role in mitotic exit and cytokinesis, microtubules have gained significant interest as important target of cancer therapy (Tangutur et al., 2017). In the present study, microtubule-associated protein TPX2 was highly expressed in ccRCC. TPX2 (Targeting protein for Xenopus kinesin-like protein 2) is required for microtubule formation in human cells. As a critical regulator of mitosis, TPX2 cooperates with Aurora-A kinase and Eg5 kinesin to control microtubule assembly and spindle stability. Several studies have demonstrated that TPX2 is overexpressed in esophageal squamous cell carcinoma, breast, colon and prostate cancer (Hsu et al., 2014; Wei et al., 2013; Yang et al., 2015; Zou et al., 2018). TPX2 was reported as a prognostic marker and potential therapeutic target in ccRCC (Glaser et al., 2017).

The multiple genomic alterations in most cancers may be linked to various DNA metabolic processes, including fidelity of the DNA synthesis and mismatch repair. In this study, hub gene TOP2A is involved in the DNA synthesis and highly expressed in ccRCC. TOP2A (Topoisomerase 2-alpha) is a critical enzyme in DNA replication, transcription and regulating the topologic states of DNA. Studies have confirmed that TOP2A is implicated in various types of tumors, such as gastric cancer (Terashima et al., 2017), colon cancer (Hou et al., 2018), pancreatic cancer (Pei, Yin & Liu, 2018) and papillary renal cell carcinoma (Ye et al., 2018). TOP2A is reported to be a novel prognostic marker in renal cell carcinoma (Lu et al., 2017).

In addition, hub gene NCAPG is highly expressed in ccRCC. NCAPG (Non-SMC condensin I complex subunit G) is a mitosis-related chromosome condensation protein, which reconstitutes long and thin chromatin strands into compact short chromosomes. The non-SMC subunits control the activity of ATP-dependent DNA supercoiling and chromosome segregation. Dysregulation of NCAPG may contribute to the progression of gastric cancer (Song et al., 2018). Overexpression of NCAPG is associated with recurrence and survival of tumor patients (Sun et al., 2018).

Fatty acid metabolic disorders are considered to be a component of malignant transformation in many cancers (Monaco, 2017). In the present study, low-expression of ACADM is involved in the ccRCC tumorigenesis. ACADM (Acyl-CoA Dehydrogenase Medium Chain) is specific for acyl chain of 4 to 16 lengths. The homotetramer ACADM catalyzes the initial step of the mitochondrial fatty acid beta-oxidation pathway. Although the molecular pathological mechanisms of low expression of ACADM in cancer remain unclear, study has shown that HIF-1 mediated suppression of acyl-CoA dehydrogenases and fatty acid oxidation is critical for cancer progression (Huang Li et al., 2014). Another study also showed that in a breast cancer transgenic mouse model, attenuating medium-chain acyl-CoA dehydrogenase activity accelerated cancer progression (Niu et al., 2017).

In the present study, the survival analyses revealed that ten up-regulated and one down-regulated hub genes were significantly correlated with worse overall survival of ccRCC patients. These genes include CENPE, KIF20A, KIF4A, MELK, NCAPG, NDC80, NUF2, TOP2A, TPX2, UBE2C and ACADM. The survival analyses indicate that these hub genes may play important roles in the carcinogenesis, progression, invasion or recurrence of ccRCC.

The pathogenesis of ccRCC is a complex process driven by specific genetic and epigenetic alterations. The discovery of new potential markers will contribute to the early diagnosis and effective treatment of ccRCC. Literature retrieval results showed that some hub genes in present study (DTL, KIF20A, KIF4A, MELK, NCAPG, NUF2, TOP2A, TPX2 and UBE2C) were consistent with previous studies (Chen et al., 2018; Wang et al., 2019; Yuan et al., 2018). Meanwhile, we found three new hub genes (NDC80, CENPE and ACADM) related to the prognosis of ccRCC. These findings may advance the understanding of the pathogenesis of ccRCC and provide novel targets for diagnosis, clinical treatment and prognosis.

Some limitations of our study should be mentioned. First, relatively few samples carry a risk of failing to demonstrate the hub genes. This may explain four candidate hub genes have no statistically significant differences in 44 pairs of specimens. Similarly, two candidate hub genes identified from chip dataset with 72 pairs of samples can’t be validated in TCGA database with 343 samples. Second, 44 pairs of samples used for hub genes validation originate from Chinese Han ethnicity, which is different from that of chip dataset samples (American population). This may be the reason why some hub genes cannot be verified. Therefore, these hub genes may need to be validated in different ethnic population.

## Conclusions

In conclusion, the present study identified 12 hub genes that may be involved in the carcinogenesis or progression of ccRCC. Among them, 11 hub genes are closely related to the prognosis of ccRCC. These hub genes may be regarded as diagnostic and prognostic biomarkers, and could become potential targets for future ccRCC therapeutic strategies. However, the function of these genes in ccRCC needs further study to elucidate the biological characteristics.

##  Supplemental Information

10.7717/peerj.8096/supp-1 File S1 Raw data of key differentially expressed genes in ccRCC

10.7717/peerj.8096/supp-2 File S2 Raw data for NDC80

## Additional Information and Declarations

### Data Availability

The following information was supplied regarding data availability: The raw data is available at NCBI GEO: GSE53757.
